# Resection of the gastric submucosal tumor (G-SMT) originating from the muscularis propria layer: comparison of efficacy, patients’ tolerability, and clinical outcomes between endoscopic full-thickness resection and surgical resection

**DOI:** 10.1007/s00464-019-07311-x

**Published:** 2020-02-03

**Authors:** Sha Liu, Xinxin Zhou, YongXing Yao, Keda Shi, Mosang Yu, Feng Ji

**Affiliations:** 1 Department of Anesthesiology, The First Affiliated Hospital, School of Medicine, Zhejiang University, No. 79 Qingchun Rd, Hangzhou, Zhejiang Province 310003 China; 2 Department of Gastroenterology, The First Affiliated Hospital, School of Medicine, Zhejiang University, No. 79 Qingchun Rd, Hangzhou, Zhejiang Province, 310003 China; 3 Department of Lung Transplant, The First Affiliated Hospital, School of Medicine, Zhejiang University, No. 79 Qingchun Rd, Hangzhou, Zhejiang Province, 310003 China

**Keywords:** Endoscopic full-thickness resection (EFTR), General surgery, Laparoscopy, Gastrointestinal stromal tumors (GIST), Lamina propria, Postoperative complications

## Abstract

**Background and aims:**

Endoscopic full-thickness resection (EFTR) has been increasingly applied in the treatment of gastric submucosal tumors (G-SMTs) with explorative intention. This study aimed to compare the efficacy, tolerability, and clinical outcomes of EFTR and surgical intervention for the management of muscularis propria (MP)-derived G-SMTs.

**Methods:**

Between September 2011 and May 2019, the clinical records of patients with MP-derived G-SMTs undergoing EFTR at our endoscopic unit were collected. A cohort of people with primary MP-derived G-SMTs treated by surgery was matched in a 1:1 ratio to EFTR group with regard to patients’ baseline characteristics, clinicopathologic features of the tumor and the procedure date. The perioperative outcomes and follow-up data were analyzed.

**Results:**

In total, 62 and 62 patients were enrolled into the surgery and EFTR group, respectively, with median follow-up of 786 days. The size of G-SMTs (with ulceration) ranged from 10 to 90 mm. For patients with tumor smaller than 30 mm, surgery and EFTR group presented comparable procedural success rate (both were 100%), en bloc resection rate (100% vs. 94.7%), tumor capsule rupture rate (0% vs. 5.3%), and pathological R0 resection rate (both were 100%). EFTR had a statistically significant advantage over surgery for estimated blood loss (3.12 ± 5.20 vs. 46.97 ± 60.73 ml, *p* ≤ 0.001), discrepancy between the pre- and postprocedural hemoglobin level (5.18 ± 5.43 vs. 9.84 ± 8.25 g/L, *p* = 0.005), bowel function restoration [1 (0–5) vs. 3 (1–5) days, *p* ≤ 0.001], and hospital cost (28,617.09 ± 6720.78 vs. 33,963.10 ± 13,454.52 Yuan, *p* = 0.033). The patients with tumor larger than 30 mm showed roughly the same outcomes after comparison analysis of the two groups. However, the clinical data revealed lower en bloc resection rate (75.0% vs. 100%, *p* = 0.022) and higher tumor capsule rupture rate (25.0% vs. 0%, *p* = 0.022) for EFTR when compared to surgery. The procedure time, duration of postprocedural fasting and antibiotics usage, and hospital stay of the two groups were equivalent. The occurrence rate of adverse events within postoperative day 7 were 74.2% and 72.6% after EFTR and surgery, respectively (*p* = 1.000). No complications occurred during the follow-up.

**Conclusion:**

For treatment of MP-derived G-SMTs (with or without ulceration), our study showed the feasibility and safety of EFTR, which also provided better results in terms of procedural blood loss, the postoperative bowel function restoration and cost-effectiveness when compared to surgery, whereas the surgery was superior in en bloc resection rate for G-SMTs larger than 30 mm. The postprocedural clinical outcomes seemed to be equivalent in these two resection methods.

Endoscopic resection maneuvers for gastrointestinal (GI) tumors have advanced substantially in recent decades. Most gastric submucosal tumors (G-SMTs) grow intraluminally and rarely metastasize to local lymph nodes, and the gastrointestinal stromal tumors (GISTs) account for a great proportion of G-SMTs [1]. For the G-SMTs originating from or infiltrating the muscularis propria (MP) layer or deeper, the endoscopic submucosal excavation (ESE) or endoscopic muscularis dissection (EMD) seems to be more suitable to be carried out than endoscopic submucosal dissection (ESD) [2]. However, perforation during resection could not be avoided especially for the G-SMTs affecting serosa and with an extraluminal component. Based on the successful management of the unavoidable perforation by metallic clips or nylon loop suturing, the ESD-derived Endoscopic full-thickness resection (EFTR) for G-SMTs treatment is technically possible and increasingly applied [3, 4].

The therapeutic potential of EFTR for GI-SMTs was introduced for the first time by Suzuki et al. in [5]. Since then, the beneficial outcome of EFTR for G-SMT treatment has been constantly reported by advanced centers in Asia [6–9]. Zhou et al. presented the first series of EFTR (without laparoscopic assistance) for G-SMT resection with 100% complete resection rate, while the postprocedural complications barely occured [1]. In China, this endoscopic technique has gradually gained acceptance in clinical practice [10].

Small G-SMTs (≤ 30 mm) without ulceration is currently considered to be eligible for EFTR [10], and the EFTR was stated as equally efficient as the laparoscopy and endoscopy cooperative surgery (LECS)-related procedures but less invasive in resection of small G-SMT [10]. It is believed by some experts that the superiority of EFTR/ESD was particularly highlighted with respect to the non-intracavitary GIST [6, 11]. However, for the undiagnosed G-SMTs or suspected primary GISTs, which are larger than 20 mm in diameter or symptomatic, the laparoscopic resection (with endoscopic assistance) is routinely indicated after ruling out the metastasis and seems potentially curative [1, 12–16]. In comparison, the evidences supporting the clinical efficacy, safety and long-term satisfactory oncological outcomes of EFTR for G-SMT resection are still lacking [9, 15]. The technically challenging EFTR currently has standardization and popularization problem, since it requires sophisticated endoscopic skills including electrosurgical incision, hemostasis and endoluminal closure of GI defects. To define the feasibility of EFTR as well as to characterize the clinical outcomes of EFTR and surgical resection for G-SMT treatment, we conducted a retrospective study to compare the EFTR and surgery for G-SMT removal.

## Patients and methods

### Patients and study design

**Fig. 1:**
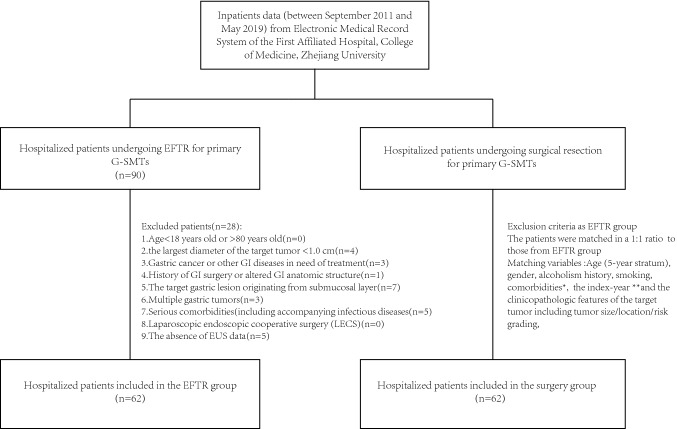
Flow chart for selecting study cohorts. *GI* gastrointestinal tract, *G-SMT* gastric submucosal tumor, *EFTR* endoscopic full-thickness resection, *EUS* endoscopic ultrasound. *Cormorbidities were already defined before the index date. **The procedure date of EFTR or surgery was defined as the index date, and the index-year was the year part of index date

This study was approved by the institutional review board (First Affiliated Hospital, School of Medicine, Zhejiang University). The clinical records of a consecutive series of 90 patients, who underwent EFTR for primary G-SMTs at the department of gastroenterology between September 2011 and May 2019, were collected. 62 patients were enrolled into the EFTR group (study group) after exclusion based on the following criteria: (1) age < 18 or > 80; (2) the largest diameter of the target tumor < 1.0 cm; (3) gastric cancer or other GI diseases requiring treatment; (4) history of GI surgery or altered GI anatomic structures; (5) target gastric lesions originating from the submucosal layer; (6) multiple gastric tumors; (7) serious comorbidities; (8) patients undergoing LECS; and (9) the absence of EUS data (Fig. 1). For comparative assessment, a group of 62 patients with G-SMTs treated by surgery in the same period was matched for patient baseline characteristics, tumor clinicopathological features as well as the year part of the procedure date (Fig. 1). Overall, there were 62 and 62 patients in the EFTR and surgery group, respectively. To comply with the research ethics and the Personal information Protection Act, the included patients were replaced with surrogate numbers when we analyzed the data.

Prior to the EFTR or surgery, all patients were examined by ultrasonography and/or computed tomography (CT) and/or magnetic resonance imaging (MRI) of the abdomen to exclude tumor metastasis. Endoscopic ultrasonography (EUS) was also performed to determine the tumor size, originating layer, internal echogenicity, and growth pattern of target G-SMTs. The patients were followed up until September 15th, 2019 or death or tumor recurrence/metastasis requiring treatment or lost to follow-up, depending on which came first.

### Procedure descriptions

EFTR, as a novel and technically demanding procedure, allows for excision of a small piece of the complete (full-thickness) GI wall by using per-oral endoscopy. This resection maneuver is considered suitable for G-SMTs smaller than 30 mm and without ulceration, while the large G-SMTs (≥ 30 mm) were generally indicated for surgical resection [12, 17, 18]. However, the present study demonstrated that some large tumors (with ulcerations) could be removed by EFTR based on thorough preprocedural examinations with the assistance of abdominal CT, MRI, and EUS. The determination of the resection method for G-SMT was based on the overall consideration of the tumor characteristics, the operators’ experience, and the patients’ preferences. Maintaining the intact tumor capsule and minimizing the injuries to the surrounding normal tissue were prioritized during the resection.

### Endoscopic full-thickness resection (EFTR)

All procedures were performed by the same team of experienced endoscopists (F.J, L.H.C., H.T.C. C.H.Y.) who had completed more than 1500 endoscopic treatment sessions within the upper GI tract, including ESD, endoscopic mucosal resection (EMR), per-oral endoscopic myotomy (POEM), Submucosal tunneling endoscopic resection (STER). The collaboration of two trained nurses or technicians and one anesthetist was also required.

**Fig. 2:**
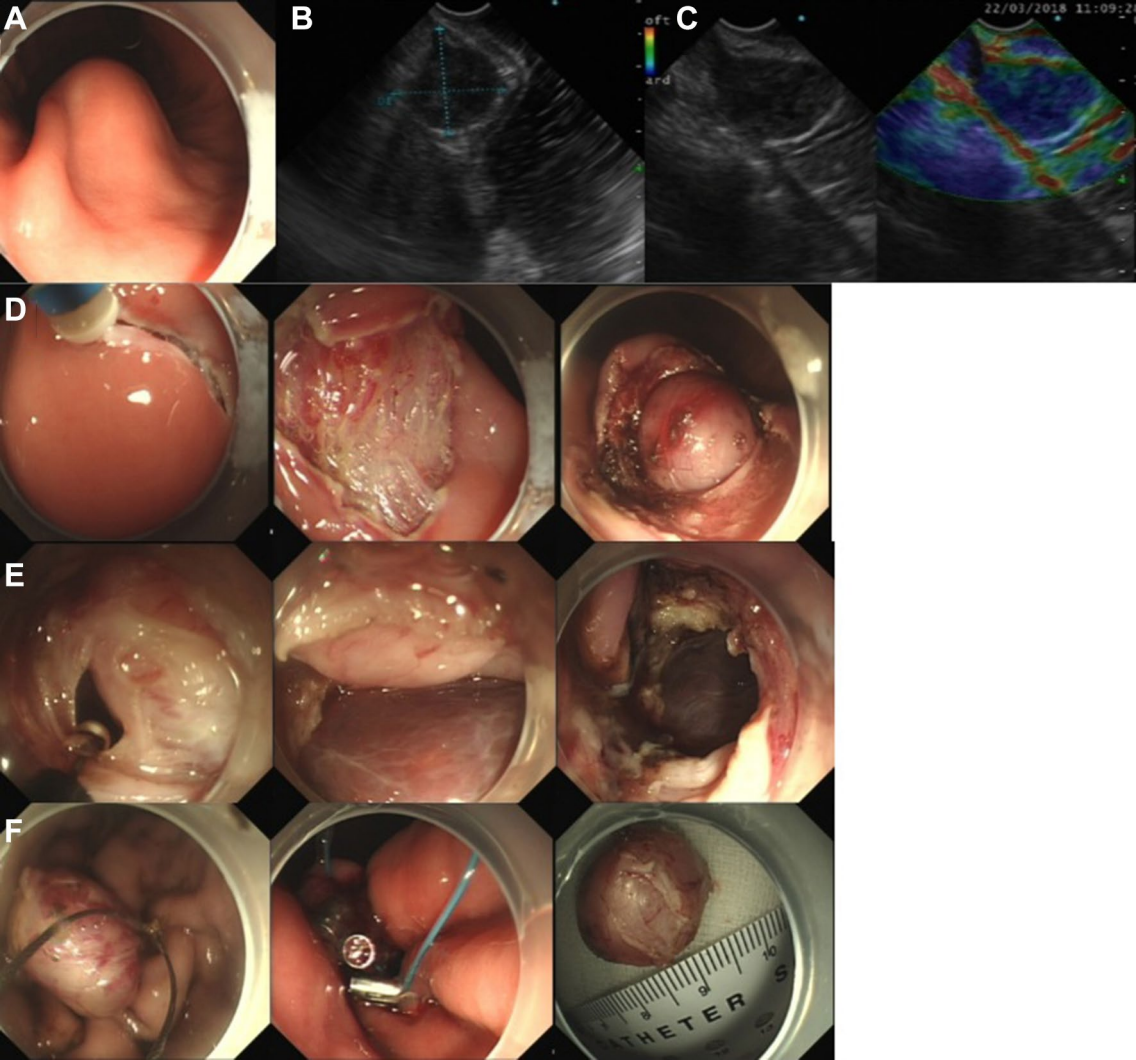
Separate maneuvers of EFTR for a muscularis propria (MP)-originating gastric submucosal tumor (G-SMT): **A** the protrusive submucosal lesion with smooth surface at the gastric greater curvature, **B**, **C** this well-demarcated and heterogeneously hypoechoic lesion showing moderate enhancement on the contrast-enhanced endoscopic ultrasound (EUS), **D** circumferential pre-cutting around the lesion and separating the submucosal tissue to expose the tumor, **E** separating the muscularis propria tissue associated with the tumor and creating an active perforation, **F** completely snaring the target tumor, closing the gastric wall defect with purse-string closure method by nylon cord and multiple metallic clips

After the patient was kept nil per os (NPO) for at least 6 h, propofol (1.5–2.0 mg/kg) was administered, whereas the general anesthetic with endotracheal intubation was selected for the anxious/agitated patients. During the whole procedure, the carbon dioxide (CO_2_) insufflation of the peritoneal cavity and a double-channel upper gastrointestinal endoscopy (GIF-2TQ260M, Olympus, Tokyo, Japan) were applied. The basic operative steps are as follows: (1) marking dots were pointed circumferentially 2 mm away from margin of the target lesion with an electrosurgical knife; (2) a mixture solution (0.9% normal saline/10% glycerin fructose plus epinephrine (1:10,000) plus indigo carmine) was injected into the submucosal layer to facilitate submucosal elevation; (3) with appropriate choice of knives including Dual knife (KD-650L/U/Q, Olympus), Hook knife (KD-620LR, Olympus), and hybrid knife (20150-060/-300, Erbe Elektromedizin, Tübingen, Germany), a circumferential pre-cutting around the lesion was carried out deeply enough to gain access to the submucosal space. In some cases, the mucosa and submucosa covering the lesion are dissected in order to assess the tumor margin and to improve the operation view; (4) the MP tissue associated with the lesion was separated by hybrid knife or the alternate cutting with insulated-tip (IT) knife (KD-611L, Olympus) and Hook/Dual knife; (5) after the lesion was almost exposed and the remnant attached tissue was less than one-fifth, the lesion with the surrounding gastric tissue was resected by snaring method (NOE 342216-G, ENDO-FLEX, Voerde, Germany; M00562670, Boston Scientific Corporation, Natik, USA), and an active perforation would be created; (6) the gastric wall defect was approximated and closed by metallic clips (M00522610, Boston Scientific Corporation), or purse-string suturing method (loop-and-clip closure technique) using metallic clips and endoloops (LeCamp™ Loop-20/-30, LeoMed, Changzhou, China). For larger defects, the over-the-scope-clip (OTSC, 100.12, Ovesco Endoscopy, Tübingen, Germany) could also be opted (Fig. 2) [10]; (7) the lesion was pulled towards the gastric cavity and retrieved through the mouth; (8) a 20-gauge needle was inserted into the right upper quadrant to relieve the pneumoperitoneum; and (9) a nasogastric (NG) tube was routinely placed for GI decompression.

### Surgical resection

For G-SMT resection, either the laparoscopic wedge resection or the extended resection (e.g., total gastrostomy) is preferred depending on the size and anatomic site of the G-SMT [19]. The target gastric lesion at the gastric body or fundus was managed with wedge or sleeve-type resection. The localized lesion along with the associated gastric tissue was dissected using the linear gastrointestinal stapler (s) (EC60, Johnson & Johnson, New Jersey, USA) or the electrocautery device with a surgical margin of 10–20 mm, and then, it was put into a specimen bag and exteriorized through the trocar. The gastric wall defects were closed with a linear stapler(s) or hand-sewn. For larger lesions (≥ 50 mm) or lesions at specific locations (proximal to or at the gastroesophageal junction or cardia or antrum) [18, 20], the subtotal or distal gastrectomy with gastrojejunal or gastroduodenal reconstruction would be recommended [20].

A thorough screening for metastatic signs in the abdomen was also required during the surgery. Any suspicious lesions on the peritoneum would be resected (if possible) or biopsied for further staging of the tumor. The resection of any enlarged lymph nodes along with the primary lesion was also pursued. For the tumors with extension or invading into the adjacent organs, a more invasive resection should be performed to ensure tumor en bloc resection.

### Specimen preparation and pathological analysis

The specimen was cut open along the suture lines and measured with a millimeter ruler. Then it was immersed in 10% formalin solution. The sectioned tumor slices were stained with hematoxylin and eosin (H&E), and the number of mitotic figures per 50 high-powered fields was counted. The gastric GIST was confirmed by the immunohistochemical analysis of CD-117 (c-KIT), CD-34, DOG-1, SMA, Desmin, S-100 along with genotyping of KIT or PDGFRA. The risk classification of GISTs referred to the revised NIH grading system by Joensuu [21].

### Postoperative management

Patients were kept NPO for at least 48 h until occurrence of anal exsufflation or withdrawal of the NG tube. Then the patients were on liquid diets for the following 2–3 days and gradually returned to a normal diet over 1 week. Antibiotics (piperacillin–tazobactam 4.5 g or ceftriaxone 2 g three times daily plus ornidazole 500 mg twice daily), proton pump inhibitors (PPI, esomeprazole or pantoprazole 40 mg twice daily), hemostatics were administered intravenously. Then the PPI medications were orally taken for another 4 weeks after patient discharge.

### Definitions

(1) *Tumor size* The size was determined as the length in the largest dimension of the resected tumor. (2) *Procedural success* The target G-SMT was completely resected (en bloc or in piecemeal) by EFTR or surgery (laparoscopy or open surgery), and the gastric wall defect was successfully closed during the same procedure. (3) *Histological confirmed R0 resection* The dissected specimen without tumor residue at the resection margin was confirmed based on the histological examination. (4) *Blood loss* During the whole resection process, the amount of bleeding was estimated by the volume of blood collected in the suction tank and/or weighing the gauze swabs collecting the blood.

### Follow-up

The follow-up strategy depended on pathological reports of the G-SMT. Approximately 1 month after G-SMT resection by EFTR, the GI endoscopy was conducted to observe postprocedural healing and exclude residual tumors. The surveillance endoscopy was then recommended for the patients every 3–6 months, and the re-examination interval could be prolonged to 1 year after 3 endoscopic sessions. The patients who underwent surgery received first endoscopy at 3 months postoperatively and then repeated semiannually. All patients were periodically (every 6 months–1 year) followed up through an outpatient visit or telephone call to monitor their oncological outcomes. The re-examination records of endoscopy, EUS, CT, or MRI were collected and reviewed.

### Statistical analysis

The SPSS 19.0 statistical software (SPSS, Inc., Chicago, IL, USA) was used to set up the database and analyze the results. Measured data were expressed as the mean value ± standard deviation (SD). Differences between two mean values were estimated by an unpaired Student *t* test. The categorical variables were presented as frequencies and corresponding percentages, and the Pearson Chi-square or Fisher’s exact test was used for comparison. The parameters relative to number of days (follow-up duration, hospital stay, postoperative bowel function restoration, fasting, antibiotics usage) were evaluated using the Wilcoxon Mann–Whitney test and quantified by the median with range. All statistical tests were two-sided, and *p* < 0.05 was considered significant.

## Results

### Patients’ baseline characteristics and clinicopathological features of the target G-SMTs

62 patients underwent G-SMT resection utilizing EFTR method (59.7% female, 57.35 ± 9.88 years of age). The pair-matching yielded a group of 62 patients receiving surgical resection for G-SMTs, who were comparable to the EFTR group in terms of 5 principle patient baseline parameters (age, gender alcohol drinking, smoking, comorbidities) and 5 main G-SMT features (tumor size, location, mucosal erosion or ulceration, organ invasion, pathological type). There were no significant inter-group differences after matching. The mean sizes of the G-SMTs in EFTR and surgery group were 28.16 ± 15.23 mm and 27.97 ± 15.46 mm, respectively, meanwhile, the proportions of the tumors smaller than 30 mm in both groups were statistically similar. The gastric body and fundus accounted for the major part (84.68%) of tumor resection sites. In the EFTR group, 10 (16.1%) cases with erosive or ulcerative G-SMTs were observed, whereas there were 6 similar cases in the surgery group. Besides, the relative proportion of the gastric GISTs to the non-GISTs (96.8% to 3.2%) as well as the distributions of GIST risk classification in both groups were comparable. In surgical resection group, 2 non-GISTs were schwannoma and leiomyoma as determined by histological examination, while 2 non-GISTs in the EFTR group turned out to be schwannomas. The patient demographics and target G-SMT features are detailed in Tables 1 and 2.

**Table 1:** Patients’ demographic and baseline characteristics of both groups and the matching results

	Surgery (*n* = 62)	ETFR (*n* = 62)	*p* value
Age, mean ± SD, years	58.92 ± 9.66	57.35 ± 9.88	0.374
Gender, *n* (%)			
Female	38 (61.3)	37 (59.7)	1.000
Male	24 (38.7)	25 (40.3)	
Alcohol drinking, *n* (%)^a^	11 (17.7)	13 (21.0)	0.821
Cigarette smoking, *n* (%)	5 (8.1)	10 (16.1)	0.270
Comorbidities, *n* (%)			
Overall	39 (62.9)	36 (58.1)	0.714
Hypertension	29 (46.8)	21 (33.9)	0.200
Diabetes mellitus	6 (9.7)	10 (16.1)	0.422
Upper GI ulcers	6 (9.7)	11 (17.7)	0.296
Hepatitis^b^	3 (4.8)	2 (3.2)	1.000
Heart disease^c^	5 (8.1)	3 (4.8)	0.717
CVD^d^	0 (0)	3 (4.8)	0.244
Respiratory diseases^e^	5 (8.1)	1 (1.6)	0.207
Renal disease	0 (0)	1 (1.6)	1.000

*EFTR* endoscopic full-thickness resection, *GI ulcers* gastrointestinal ulcers, *CVD* cerebrovascular disease, *SD* standard deviation

^a^Alcohol-related diseases or excessive alcohol drinking

^b^Chronic hepatitis B

^c^Sinus bradycardia with arrhythmia, cardiac premature beat, atrial fibrillation, coronary heart disease

^d^History of the cerebral infarction, cerebral hemorrhage

^e^Pneumonia, chronic bronchitis, bronchiectasia, silicosis with cor pulmonale, pulmonary nodules

**Table 2:** G-SMTs’ clinicopathologic characteristics of both groups and the matching results

	Surgery (*n* = 62)	ETFR (*n* = 62)	*p* value
Tumor size			
Mean ± SD, mm	27.97 ± 15.46	28.16 ± 15.23	0.944
< 30 mm			
*n* (%)	38 (61.3)	38 (61.3)	1.000
Mean ± SD, mm	19.00 ± 5.02	19.26 ± 5.26	0.824
≥ 30 mm			
*n* (%)	24 (38.7)	24 (38.7)	1.000
Mean ± SD, mm	42.17 ± 15.81	42.25 ± 15.25	0.985
Tumor location, *n* (%)			
Gastric body	36 (58.1)	35 (56.5)	0.977
Fundus	16 (25.8)	18 (29.0)	
GEJ or cardia	7 (11.3)	7 (11.3)	
Anturm	3 (4.8)	2 (3.2)	
Mucosal erosion or ulceration, *n* (%)	6 (9.7)	10 (16.1)	0.422
Adjacent organs invasion, *n* (%)	3 (4.8)	1 (1.6)	0.619
Pathological type, *n* (%)			
Non-GIST	2 (3.2)	2 (3.2)	1.000
GIST	60 (96.8)	60 (96.8)	
Risk grading			
Very low	7 (11.7)	15 (25.0)	0.164
Low	37 (61.7)	36 (60.0)	
Intermediate	14 (23.3)	8 (13.3)	
High	2 (3.3)	1 (1.7)	

*G-SMT* gastric submucosal tumor, *EFTR* endoscopic full-thickness resection, *GEJ* gastroesophageal junction, *GIST* gastrointestinal stromal tumors, *SD* standard deviation

### Perioperative outcomes

The operation-related data for EFTR and surgery group were analyzed in subgroups of different tumor size ranges. For the patients with G-SMTs smaller than 30 mm, both groups presented 100% procedural success rate and R0 resection rate; however, 2 patients experienced piecemeal resection of the target G-SMTs by EFTR. There were no significant disparity between these two resection methods regarding procedural duration, blood transfusion rate (both were 0%), duration of postoperative fasting and antibiotics usage, hospital stay, with the exception of blood loss, which was significantly lower in the EFTR group (3.12 ± 5.2 ml vs. 46.97 ± 60.73 ml, *p* ≤ 0.001), restoration of the bowel function [1 (0–5) days vs. 3 (1–5) days, *p* ≤ 0.001], and hospitalization cost (28,617.09 ± 6720.78 vs. 33,963.10 ± 13,454.52 Yuan, *p* = 0.033) (Table 3a). For the patients with G-SMTs larger than 30 mm, the en bloc resection rate of G-SMT decreased significantly in the EFTR group, which was 75%, while the tumor capsule rupture rate increased correspondingly, which was 25%. In comparison, the surgery yielded a 100% of success rate in complete tumor resection, and the difference was significant (*p* = 0.022). In addition, the EFTR failed in 1 case involving a G-SMT with size of 90 × 30 mm, which resulted in an immediate transference to laparoscopic closure of the large gastric perforation caused during EFTR. The rate of histological R0 resection in two groups reached 100%. Besides, EFTR was still associated with less intraoperative bleeding volume (5.67 ± 11.29 vs. 52.50 ± 36.30 mL, *p* < 0.001), sooner bowel function restoration after procedure [1 (0–6) vs. 3 (2–7) days, *p* < 0.001], and less cost (32,661.11 ± 11,529.66 vs. 41,981.29 ± 14,911.18 Yuan, *p* = 0.019). The other operative parameters were statistically comparable between the two groups (Table 3b).

**Table 3:** Perioperative data of the EFTR and surgical resection for G-SMTs (a) (< 30 mm), (b) (≥ 30 mm)

(a)	Surgery (*n* = 38)	ETFR (*n* = 38)	*p* value
Procedural success rate, *n* (%)	38 (100)	38 (100)	ND
Tumor capsule rupture, *n* (%)	0 (0)	2 (5.3)	0.493
En bloc resection, *n* (%)	38 (100)	36 (94.7)	0.493
Histological R0 resection, *n* (%)	38 (100)	38 (100)	ND
Intraoperative bleeding			
Total volume, mean ± SD, mL	46.97 ± 60.73	3.12 ± 5.20	< 0.001
Δ(pre-HB)-(post-HB), mean ± SD, g/L	9.84 ± 8.25	5.18 ± 5.43	0.005
Blood transfusion rate, *n* (%)	0 (0)	0 (0)	ND
Procedure duration, mean ± SD, min	100.66 ± 44.44	108.84 ± 78.02	0.576
Postoperative bowel function restoration, median (range), days	3 (1–5)	1 (0–5)	< 0.001
Postoperative fasting, median (range), days	4 (1–7)	4 (2–7)	0.512
Postoperative antibiotics usage, median (range), days	5 (2–10)	5 (3–7)	0.903
Hospital stay, median (range), days	12 (4–33)	11 (6–20)	0.194
Hospitalization expenses, mean ± SD, yuan	33,963.10 ± 13,454.52	28,617.09 ± 6720.78	0.033

(b)	Surgery (*n* = 24)	ETFR (*n* = 24)	*p* value
Procedural success rate, *n* (%)	24 (100)	23 (95.8)	1.000
Tumor capsule rupture, *n* (%)	0 (0)	6 (25.0)	0.022
En bloc resection, *n* (%)	24 (100)	18 (75.0)	0.022
Histological R0 resection, *n* (%)	24 (100)	24 (100)	ND
Intraoperative bleeding			
Total volume, mean ± SD, ml	52.50 ± 36.30	5.67 ± 11.29	< 0.001
Δ(pre-HB)-(post-HB), mean ± SD, g/L	8.83 ± 10.31	2.42 ± 5.52	0.011
Blood transfusion rate, *n* (%)	1 (4.2)	0 (0)	1.000
Procedure duration, mean ± SD, min	130.96 ± 63.30	127.42 ± 62.48	0.846
Postoperative bowel function restoration, median (range), days	3 (2–7)	1 (0–6)	< 0.001
Postoperative fasting, median (range), days	4 (2–27)	4 (1–10)	0.075
Postoperative antibiotics usage, median (range), days	5.5 (3–16)	4 (2–11)	0.104
Hospital stay, median (range), days	11.5 (8–29)	11.5 (7–24)	0.462
Hospitalization expenses, mean ± SD, yuan	41,981.29 ± 14,911.18	32,661.11 ± 11,529.66	0.019

*EFTR* endoscopic full-thickness resection, *G-SMT* gastric submucosal tumor, *Pre-HB* preoperative hemoglobin level, *Post-HB* postoperative hemoglobin level, *SD* standard deviation

### Complications within 7 days postoperatively and long-term follow-up

The follow-up was longer for the surgery group than for the EFTR group [1089 (110–3282) vs. 740 (120–2964) days, *p* = 0.013]. In total, 91 out of 124 patients developed the procedure-related complications, which were mostly relieved with or without medication treatments. The occurrence rates of bradycardia, hypoxia, hypotension, abdominal distention, nausea, chest pain, fever, GI bleeding, or perforation in two groups were statistically similar. The abdominal pain (38.7%) and laryngopharyngeal discomfort (25.8%) appeared more often in patients receiving EFTR (both were *p* ≤ 0.01) (Table 4), but there was higher likelihood of having cough or expectoration after surgery (33.9% vs. 8.1%, *p* = 0.001) (Table 4). 3 patients in the two groups experienced blood loss (< 100 mL) during the early postoperative period, which was successfully stopped by irrigation of norepinephrine and thrombin through the NG tube. There were two cases in the EFTR group involved with procedure-related GI perforation. In one case, the patient suffered from intolerable pharynx and substernal pain caused by minor esophageal perforation owing to a large tumor (60 × 50 mm) retrieval, and the endoscopic management for this perforation was needed. In another case, a minor perforation at the resection site healed after conservative treatments. The delayed GI bleeding or perforation, tumor recurrence or metastasis, death were not observed in both groups during the long-term follow-up (Table 4). One patient in the surgery group developed esophageal anastomosis stenosis 10 months after proximal subtotal gastrectomy for G-SMT, which required rehospitalization for endoscopic dilation with Savary–Gilliard bougies (not shown).

**Table 4:** Clinical outcomes of the EFTR and surgical resection for G-SMTs during follow-up

	Surgery (*n* = 62)	ETFR (*n* = 62)	*p* value
Follow-up, median (range),days	1089 (110–3282)	740 (120–2964)	0.013
Within 7 days postoperatively, *n* (%)			
Overall	45 (72.6)	46 (74.2)	1.000
Bradycardia^a^	1 (1.6)	1 (1.6)	1.000
Hypoxia^b^	0 (0)	1 (1.6)	1.000
Hypotension^c^	1 (1.6)	4 (6.5)	0.365
Abdominal pain	10 (16.1)	24 (38.7)	0.008
Abdominal distention	6 (9.7)	8 (12.9)	0.778
Nausea	14 (22.6)	9 (14.5)	0.356
Cough/expectoration	21 (33.9)	5 (8.1)	0.001
Laryngopharyngeal discomfort	1 (1.6)	16 (25.8)	< 0.001
Retrosternal chest pain	0 (0)	2 (3.2)	0.496
Fever^d^	18 (29.0)	13 (21.0)	0.407
Bleeding^e^	2 (3.2)	1 (1.6)	1.000
Perforation	0 (0)	2 (3.2)	0.496
After 7 days postoperatively, *n* (%)			
Bleeding^f^	0 (0)	0 (0)	ND
Perforation	0 (0)	0 (0)	
Metastasis	0 (0)	0 (0)	
Recurrence	0 (0)	0 (0)	
Death	0 (0)	0 (0)	

*G-SMT* gastric submucosal tumor, *EFTR* endoscopic full-thickness resection, *ND* no difference

^a^Resting heart rate less than 50 beats/min, which was determined either by palpation or electrocardiography

^b^Blood oxygen desaturation less than 85% on pulse oximetry

^c^The systolic blood pressure was less than 90 mmHg, or blood pressure transiently dropped more than 20% from baseline (one of which was less than systolic blood pressure level of 90 mmHg), and the low blood pressure sustained for more than 15 min

^d^A temperature which was greater than 38.3 °C for more than 3 days or greater than 39.0 °C for more than 2 days

^e^Hematemesis, coffee-ground vomitus, hematochezia, or melena

^f^GI bleeding relative to the resection site requiring further hemostatic treatment, which caused an increasing pulse rate over 100 beats/min and decreasing blood pressure below 90 mmHg after a 24-h period of stable vital signs and hemoglobin level

## Discussion

To the best of our knowledge, this is the first comparison study with the largest number of included subjects to explore the clinical outcomes of EFTR and surgical resection for G-SMTs. The characteristics of patient and target tumor were specifically matched between the EFTR group and surgery group.

In the clinical practice, the G-SMTs are incidentally found by endoscopy for other reasons, such as GERD or GI ulcer, and the tumors are mostly small (< 20 mm) [14]. The included patients in our study, for example, developed symptoms including regurgitation, acid reflux, early satiety, which seemed to be relative to GI functional disorders. Considering the small G-SMTs tend to exhibit benign clinical behavior and rarely lead to metastatic disease or death, the necessity of the tumor resection remains a pending issue at present [14, 22–24]. In light of the potential malignance of GISTs [24], which account for the majority of G-SMTs, the European Society for Medical Oncology (ESMO) group proposed resection for all histologically diagnosed small GISTs [2]. However, the recent American Society for Gastrointestinal Endoscopy (ASGE) [25] and National Comprehensive Cancer Network (NCCN) guidelines [19] stated that asymptomatic tumors smaller than 20 mm without high-risk features (irregular border, cystic spaces, internal heterogeneity, hyperechoic internal echoes, ulceration, tumor progression during follow-up) could be followed up with EUS, radiological modalities, or laparoscopy. The reality is that the problems, such as patient’s low compliance with surveillance, mental strain of delayed diagnosis of malignancy, issue with cost-effectiveness, are inevitably confronted in decision-making of tumor resection [26]; therefore, resection of small G-SMTs would mostly be chosen after thorough oncological evaluation as well as considering patient’s inclination to tumor removal. Furthermore, it is still controversial and less acceptable for the small lesions (< 20 mm) to be treated by surgery [20], since the lymphadenectomy is so far not required and the surgery may overtly cause unnecessary injuries to the normal perigastric tissues and anatomical damages [27].

According to our study, the EFTR was successfully performed for the G-SMTs larger than 50 mm with 100% rate of complete resection with tumor-free margins. It seemed to be reliable to resect MP-originating tumor by EFTR, which provided quite a definite histological diagnosis of the resected specimen, consistent with previous study [15, 28, 29]. However, EFTR was not preferred as the first-line treatment for GISTs according to the previous studies and guidelines [14], especially for those with large size (> 30 mm), extraluminal component and ulceration [15]. As a supplement, the result of our study suggested that EFTR was inferior to surgical resection in terms of en bloc resection rate of G-SMTs larger than 30 mm. Some experts believed that EFTR may be associated with higher likelihood of tumor cell seeding upon pseudocapsule injury and transluminal exposure during the procedure [14, 16]; in comparison, the laparoscopy seems to be a more satisfactory resection method of G-SMTs with exophytic growth part missed or underestimated by preoperative examination [2, 19, 20, 30]. However, in the present study, the EFTR was associated with less blood loss, comparable procedure time as well as lower hospital cost relative to surgery, and neither our study nor the previous clinical reports showed the tumor occurrence or metastasis after EFTR during the long-term follow-up [1, 15, 31]. Regarding the cost-effectiveness and less invasiveness of EFTR, the widespread implementation of this endoscopic technique and its extended indications for G-SMTs are still promising.

The tumor size has been widely accepted as a main refinement for EFTR adoption in G-SMT resection. The lesions > 4 cm remain challenging with any endoscopic approach [32]. One patient in the EFTR group was transferred to laparoscopy during the procedure due to the large post-EFTR gastric wall defect caused by tumor (90 × 30 mm) resection. The in-time closure of gastric perforation by laparoscopy is exceptional emergent and essential for EFTR, since it prevents patient from postoperative peritonitis and remedial surgery after then. The importance of a combined endoscopic and laparoscopic approach in overcoming the challenges (including perforation) encountered during endoscopic approaches is also emphasized by the newest ASGE guideline [32]. For small perforations, conservative treatments including longer diet suspension, GI decompression and intravenous antibiotics is appropriate and adequate. The potential indications for large G-SMTs to be treated by EFTR require further clinical investigations, owing to the absence of well-established green channels for emergent surgery in many chinese endoscopic units.

Among the available closure and hemostasis devices for the post-EFTR defects, the metallic endoclips are the most widely applied [33]. Our study presented that the loop-and-clip closure technique (combination of metal clips with nylon snare) could effectively close the minor gastrointestinal post-EFTR defect and greatly contributed to the symptom relief as well as rapid defect healing. The endoscopist’s skill might be a more influencing factor than the perforation site in effective closure with endoclips [1]. For post-EFTR defects larger than 2 cm, the endoscopic purse-string suture (EPSS) method using endoloops and metallic clips is a rational option [8, 34–36]. The closed resection site would generally heal approximately 1 month after the procedure [33]. Our study revealed the high success rate the EPSS method obtained for post-EFTR gastric wall defect (with the maximal diameter of 70 mm) closure. The over-the-scope-clips (OTSCs), previously reported to be safe and technically reasonable for lesions ≤ 20 mm, were also used in one case with gastric wall defect approaching 40 mm in our study [37–39]. However, the OTSC-assisted EFTR has not been widely covered by Chinese health insurance, which limits its standardization and implementation. With regard to the large luminal GI defects at all thickness levels, another novel endoscopic stitching devices OverStitch™ is now superior than other counterparts in tissue approximation and suture placement [37, 40, 41]. Even though, the Overstitch’s safety and viability were only supported by minimal data concerning EMR, ESD, and EFTR [42–44]. It is obvious that the developments of the closure devices lead to simultaneous improvement of EFTR success rate.

During the G-SMT resection, massive blood loss was not observed in both resection groups; nevertheless, surgery generated significantly more blood loss than EFTR (46.97 ± 60.73 mL vs. 3.12 ± 5.2 mL, *p* < 0.001), similar to the a previous study [15]. 1 patient required blood transfusion in the surgery group. From the anatomic perspective, the gastric anterior/posterior wall side is the unfavorable location for direct intragastric performance via endoscopy (e.g., scope manipulation and operating angle) [15]; however, it is the good candidate for surgery. It is worth noting that the anterior/posterior wall side lacks the omentum preventing perigastric and intramuscular vessels and innervating nerves from incision, which may partly explain more blood loss in the surgery group. As to laparoscopic operation on lesions located in the lesser curvature side, except for the technical difficulties at this site, the resection of the omentum attachment in this excision area could also result in more intraoperative bleeding [45]. Moreover, the subtotal gastrectomy chosen for tumors at or near the gastric inlet/outlet site would cause more bleeding than other resection sites.

In the present study, the length of follow-up for surgery and EFTR group reached up to 3282 and 2964 days, respectively (*p* = 0.013), during which the major adverse events (tumor recurrence, metastasis, delayed perforation/bleeding) were entirely absent. Previous studies showed comparable postoperative complication rates between the endoscopic and laparoscopic resection methods for G-SMTs [15, 31]. In our study, no severe post-EFTR complications were reported, similar to the previous publications [1, 6]. Although there were two patients in surgery group and one patient in EFTR group involving minor bleeding within 3 days postoperatively, these patients were treated conservatively with medications and relieved without re-endoscopic management. Moreover, the abdominal pain and laryngopharyngeal discomfort occurred more often after EFTR relative to surgery (38.7% vs. 16.1%, *p* = 0.008 and 25.8% vs. 1.6%, *p* ≤ 0.001), which mostly attributed to the intraluminal resection maneuvers by the double-channel endoscopy. However, the EFTR did not give rise to the postoperative cough or expectoration.

This study has limitations. For one hand, it was a retrospective review of the data from one tertiary referral center with limited sample size. Although a case-matched comparison of EFTR and surgery for G-SMT resection was conducted, the selection bias probably existed as the resection approach was determined by the primary doctor based on tumor’s clinical characteristics as well as doctors’ preference (i.e., skills experience). For another, the follow-up interval was not long enough to determine the exact long-term results. A large, multicenter, prospective, and randomized controlled trial should be designed to enhance the statistical power and generalize the results, and in particular, a long-term assessment of oncological outcomes is necessary.

Despite the limitations, EFTR appears to be a feasible, effective, safe, and well-tolerable treatment alternative to surgery when treating MP-originating G-SMTs (with ulceration). The cost-effective quality of the EFTR has its merits for reducing financial burdens of the patients. It is undeniable that the surgical resection tends to have superiority in high en bloc resection rate and low tumor capsule rupture rate especially of large G-SMTs (≥ 30 mm), which might allow more precise pathological evaluation of the resected tumor and facilitate the follow-up strategy formulation. We suggest a cautious application of our results when translated to the general field of the G-SMTs.
